# Comparative performance analysis of neoepitope prediction algorithms in head and neck cancer

**DOI:** 10.3389/fimmu.2025.1494453

**Published:** 2025-03-04

**Authors:** Leila Y. Chihab, Julie G. Burel, Aaron M. Miller, Luise Westernberg, Brandee Brown, Jason Greenbaum, Michael J. Korrer, Stephen P. Schoenberger, Sebastian Joyce, Young J. Kim, Zeynep Koşaloğlu-Yalçin, Bjoern Peters

**Affiliations:** ^1^ Center for Infectious Disease and Vaccine Research, La Jolla Institute for Immunology, La Jolla, CA, United States; ^2^ Department of Chemistry and Biochemistry, University of California, San Diego, San Diego, CA, United States; ^3^ Division of Hematology and Oncology, UCSD Moores Cancer Center, San Diego, CA, United States; ^4^ Department of Otolaryngology-Head and Neck Surgery, Vanderbilt University, Nashville, TN, United States; ^5^ Department of Pathology, Microbiology and Immunology, Vanderbilt University Medical Center, Nashville, TN, United States; ^6^ Department of Veterans Affairs, Tennessee Valley Healthcare System, Nashville, TN, United States; ^7^ Global Clinical Development, Regeneron Pharmaceuticals, Tarrytown, NY, United States; ^8^ Department of Medicine, University of California, San Diego, San Diego, CA, United States

**Keywords:** cancer, neoepitope prediction, neoepitope screening, bioinformatics, immunogenicity

## Abstract

**Background:**

Mutations in cancer cells can result in the production of neoepitopes that can be recognized by T cells and trigger an immune response. A reliable pipeline to identify such immunogenic neoepitopes for a given tumor would be beneficial for the design of cancer immunotherapies. Current methods, such as the pipeline proposed by the Tumor Neoantigen Selection Alliance (TESLA), aim to select short peptides with the highest likelihood to be MHC-I restricted minimal epitopes. Typically, only a small percentage of these predicted epitopes are recognized by T cells when tested experimentally. This is particularly problematic as the limited amount of sample available from patients that are acutely sick restricts the number of peptides that can be tested in practice. This led our group to develop an in-house pipeline termed Identify-Prioritize-Validate (IPV) that identifies long peptides that cover both CD4 and CD8 epitopes.

**Methods:**

Here, we systematically compared how IPV performs compared to the TESLA pipeline. Patient peripheral blood mononuclear cells were cultured *in vitro* with their corresponding candidate peptides, and immune recognition was measured using cytokine-secretion assays.

**Results:**

The IPV pipeline consistently outperformed the TESLA pipeline in predicting neoepitopes that elicited an immune response in our assay. This was primarily due to the inclusion of longer peptides in IPV compared to TESLA.

**Conclusions:**

Our work underscores the improved predictive ability of IPV in comparison to TESLA in this assay system and highlights the need to clearly define which experimental metrics are used to evaluate bioinformatic epitope predictions.

## 1 Introduction

Somatic mutations in cancer cells can generate neoepitopes that are distinct from self and can be recognized by neoepitope specific-T cells in order to initiate downstream anti-tumor immune responses (1). Personalized neoepitope-based vaccines have been developed in order to leverage the anti-tumor properties of said T cells and have shown broad efficacy in a number of studies (2–8). A major limitation in the development of personalized cancer vaccines is the challenging nature of the identification of immunogenic neoepitopes (9). Somatic mutations are present in both cancer cells and cells within normal tissues. Additionally, not all somatic mutations yield epitopes that can be successfully recognized by T cells (10–12). As a result, a major area of research has been the development of tools to predict the immunogenic neoepitopes that can be generated by somatic mutations within patients’ tumors (12–17).

Several groups have developed computational pipelines that harness sequencing data from cancer patients to predict the neoepitopes that can elicit an immune response, i.e. immunogenic neoepitopes (13, 15, 18–20). Typically, these pipelines input RNA and/or exome sequencing data to identify the somatic variants that are present in a tumor. Following this, the identified variants are translated into peptides and ranked based on pipeline-specific metrics to generate a list of neoepitope candidates (21).

For a neoepitope to generate a T cell response, it must be presented by an HLA molecule and recognized by a T cell. Because of this, prediction pipelines often rank neoepitope candidates based on their potential to be presented by the specific HLA molecules present in the patient. Additionally, CD8 T cells are the primary subset of T cells that are thought to recognize neoepitopes and exert anti-tumor effects (22). As a result of this, neoepitope prediction pipelines typically focus on generating HLA class I peptides which are 8-12 amino acid residues in length (23). A prominent example of this is the workflow proposed by the Tumor Neoantigen Selection Alliance (TESLA) (19). TESLA is a consortium that evaluated a number of different neoepitope prediction tools and assessed features associated with neoepitope immunogenicity. The best performing pipelines in this study were those that placed an emphasis on features associated with peptide presentation and recognition. As a result, TESLA proposed neoepitope selection criteria which include thresholds for HLA binding affinity, abundance of the mutation in the tumor, binding stability, agretopicity (the ratio of the mutant peptide’s HLA binding affinity to wild-type peptide’s binding affinity), and foreignness (19).

Because current pipelines like TESLA only consider CD8 T cell peptides and generate a large amount of peptide candidates that cannot reasonably be tested in the limited samples typically available to us in practice, our group developed Identify-Prioritize-Validate (IPV), our in-house neoepitope prediction pipeline (24). IPV identifies the somatic variants that are highly expressed within a tumor sample using exome and RNA sequencing. Unlike other current prediction tools, IPV does not take into consideration the HLA typing of a patient, nor does it attempt to generate predictions of exact minimal epitope sequences.

Here, we assessed the ability of the IPV pipeline to identify tumor specific immunogenic peptides – regardless of the HLA restriction or CD4/CD8 phenotype – in a practical setting with limited blood volumes from patients. Additionally, we compared the performance of IPV to the TESLA pipeline. TESLA serves as an example of the metrics that are routinely being applied by current epitope prediction tools and was thus selected as the baseline comparator to IPV (25). We generated epitope predictions from tumor samples from a cohort of 11 patients with Head and Neck Squamous Cell Carcinoma (HNSCC) using the TESLA and the IPV pipelines and assessed the immunogenicity of the candidates *in vitro* with patient-matched peripheral blood mononuclear cells (PBMC). We found that in this setting, peptide candidates identified by IPV were found to be immunogenic more often than those identified by the TESLA pipeline. We validate the performance of IPV in a second cohort of patients with HNSCC and identify additional neoepitope-specific T cell responses. We have identified and discussed the likely underlying factors of this in this work.

## 2 Materials and methods

### 2.1 Sample collection

The following inclusion and exclusion criteria were considered for this study:

Inclusion criteria:

1. Adult patient (≥ 18 years old).
2. Immunocompetent patient.
3. Patient with squamous cell carcinoma of the head and neck who are undergoing surgical treatment for their malignancies.
4. Included subsites are oropharynx, oral cavity, larynx, and hypopharyngeal, and sinonasal.
5. Typically, these are patients who are undergoing composite resection with reconstruction with osseocutaneous free flap.
6. Subjects previously treated with radiotherapy or chemotherapy will be included in the study, unless they present with a severe immunocompromised state.

Exclusion criteria:

1. Patient with autoimmune diseases, HIV.
2. Patient with chronic use of steroids.
3. Patient with frank immunocompromised state.

Samples were collected after patients were examined and offered participation in the Vanderbilt Head and Neck Biorepository and Clinical Database. Tissue specimens were acquired at time of surgical resection or biopsy. A section of tumor was flash frozen by placing it into a previously labeled 1.5ml cryovial and then into a portable thermoflask containing liquid nitrogen (LN2). Frozen tissue was stored in a vapor-phase LN2 storage unit until use. PBMC specimens were acquired from patients using BD Vacutainer CPT tubes (BD #362761) before surgery or before the start of treatment.

### 2.2 HLA typing

Patient exome sequencing and RNA sequences were used to computationally determine each patient’s HLA genotype using the OptiType tool (26).

### 2.3 Whole-exome and RNA sequencing and prioritization of antigens

RNA was extracted from frozen squamous cell carcinoma of the head and neck using the Promega Maxwell Simply RNA kit (Promega #AS1390) and Promega Maxwell 16 nucleic acid extraction automated instrument (Promega #AS2000). RNA sequencing was performed by Novogene Corporation Inc. using the Illumina Platform PE 150. Sequence reads from the WES of the tumor and normal samples were aligned to the reference genome GRCh38 using SpeedSeq Align (RRID: SCR_000469) (27). Variants were then identified and annotated using SpeedSeq Somatic and SNPeff (RRID: SCR_005191) (27, 28). Variants were then filtered by selecting for mutations fulfilling the following criteria:

1. Tumor variant frequency ≥ 2%.
2. Nonsynonymous as defined by SNPeff in the hgvs_protein annotation.
3. Higher frequency of the variant in the tumor than the normal sample (ratio ≥ 1).
4. Variant is observed in RNA at least once.
5. Variant is present at a frequency ≤ 5% in the normal sample and ≥ 5% in the tumor sample.
6. Position is covered with at least 10 reads in both the tumor and normal sample.

Variants were then ranked based on the variant allele frequency (VAF) in RNA (descending order), gene expression levels in transcripts per million (descending order), and tumor genotype rank (ascending order), with tumors homozygous for the single-nucleotide polymorphism receiving the highest rank, homozygous for the reference allele receiving the lowest rank, and intermediate genotypes receiving a rank in between.

### 2.4 Generation of neoepitope candidates

From the prioritized set of antigens, neoepitopes were generated using the metrics from IPV or TESLA. For IPV peptides, two 20-mer peptides were generated for each somatic variant with the single nucleotide variant at position 6 and 15 of the peptide respectively (29). We elected to use 2 overlapping 20mers with the mutation at those positions to cover the majority of possible MHC class I and class II epitopes (24). To generate the corresponding IPV short peptides, NetMHCPan4.0 was used and the top predicted binders were selected (30).

As TESLA does not provide any platform to identify somatic variants, the same variants identified using the IPV method were used to generate epitopes to be ranked by TESLA. The TESLA ranking method was implemented as described in its publication (19). The identified variants were translated into all possible 8-12mers. The 8-12mers were then filtered according to the TELSA metrics (MHC binding affinity less than 68 nM, binding stability greater than 1.7 hours, tumor abundance above 10 TPM, agretopicity less than 0.1, and foreignness above 10^-16^) (19). HLA binding affinity and binding stability were determined using NetMHCPan4.0 and NetMHCstabpan, respectively (30, 31). Foreignness and agretopicity were calculated as described by TESLA (19). The TESLA long peptides were then generated by using the sequence of the IPV 20mer containing the corresponding TESLA 8-12mer. The workflow for neoepitope selection is outlined in **Supplementary Figure 1**. The neoepitope candidates selected for each pipeline can also be found in the **Supplementary Table 2**.

### 2.5 *In vitro* culture with neoepitope candidates

To screen the neoepitope candidates, patient PBMC were expanded *in vitro* with peptide pools containing the top ten neoepitope candidates per prediction method. Cryopreserved PBMC were thawed and counted, and samples with viability greater than 70% were used to screen neoepitope candidates. 2 x 10^6^ PBMC/well were plated in a 24 well plate (GenClone #25-107) in 1X RPMI medium (Fisher Scientific #11-875-093) supplemented with 5% human serum AB (Gemini Bio Products #100-512), 1% 100X Glutamax (Gibco #35-050-061), and 1% Penicillin: Streptomycin (Gemini Bio Products #400-109). PBMC were stimulated on day 0 with 5 ug/mL of a given peptide pool and subsequently fed with 10U/ml IL-2 (Prospec Bio #Cyt209) on days 4, 7, and 10. PBMC were harvested on day 14 and re-stimulated with the peptide pool of interest. DMSO stimulation was used as a negative control and PHA-L stimulation at 20 ug/mL was used as a positive control (Sigma Aldrich #431784-5MG). IFNg and IL-5 Fluorospot was utilized to assess PBMC activation by candidate peptides for the first cohort of patients and IFNg and IL-5 ELISpot was used for the second cohort.

### 2.6 IFNg and IL-5 ELISpot

ELISpot assays were performed in MultiScreenHTS filter plates (Fisher Scientific #MSBVN1B50) that had been coated with 5 ug/mL of IL-5 capture antibody (50uL/well, clone TRFK5 Mabtech #3391-3-250 diluted in PBS) and 5 ug/mL of IFNg capture antibody (50uL/well, clone 1-D1K Mabtech #3420-3-250 diluted in PBS). After the harvest on day 14, PBMC were plated on IFNg and IL5 coated ELISpot plates at a concentration of 100,000 cells/well in the same cell culture media described above. The PBMC were restimulated either with peptide pools or individual peptides for 24 hours. The final concentration of peptide pools and individual peptides was 5 ug/mL and 10 ug/mL respectively. Each ELISpot plate was washed five times with PBS containing 0.05% Tween 20 (MP Biomedicals #MO1TWEEN201) and then incubated for two hours at 37°C with anti-Hs IFNg-HRP Ab (Mabtech #3420-9H) at 1:200 dilution and anti-Hs IL5-HRP (Mabtech #3490-6-1000) at 1:1000 dilution. Next, each plate was washed six times with PBS and incubated for 1 hour with Dual Vectastain (Vector Laboratories #PK-6200) (100uL/well), followed by six washes with ddH_2_O. Each plate was then developed by adding 100ul/well of vector blue solution at room temperature for five to ten minutes followed by six washes with ddH_2_O and then adding 100uL/well of AEC substrate for ten minutes at room temperature. The reaction was stopped by rinsing thoroughly with cold tap water. After they completely dried, each ELISPOT plate was scanned and counted using an ImmunoSpot plate reader and associated software (Cellular Technologies).

### 2.7 IFNg and IL-5 fluorospot

PBMC were harvested and plated at a concentration of 100,000 cells/well in the same cell culture media defined above on Millipore MultiScreen-IP plates (Millipore Sigma #MSIPS4510) that had been coated with 5 ug/mL of IFNg antibody (clone 1-D1K Mabtech #3420-3-1000) and 5 ug/mL of IL-5 antibody (TRFK5 Mabtech #3490-3-1000). The PBMC were restimulated with peptide pools or single peptides at a concentration of 5 ug/ml for pools or 10 ug/ml for individual peptides on the fluorospot plates and left at 37°C overnight. To develop the plate, cells were removed and the plate was washed 5 times with 0.05% PBS Tween and the detection antibodies anti-IFNg 7-B6-1-BAM (Mabtech #3420-12) and anti-IL-5 5A10-WASP (Mabtech #3490-15) were added to each well of the plate at a 1:200 dilution in PBS containing 0.1% bovine serum albumin (BSA). The antibodies were incubated for 2 hours in the dark at room temperature. Following the incubation, the plates were washed again 5 times with the PBS Tween solution. The antibodies anti-BAM-490 (Mabtech #3640-2) and anti-WASP-640 (Mabtech #3640-6) were then added to each well at a 1:200 dilution in the PBS BSA solution and incubated in the dark for 1 hour at room temperature. The plates were washed 5 more times with PBS Tween and then Fluorescence enhancer-II (Mabtech #3641-F10) was added to each well at 50 uL/well and incubated for 15 minutes at room temperature. Following the incubation, the plates were left to dry. Once completely dry, the plates were read on the Mabtech IRIS.

### 2.8 Neoepitope screen analysis

Raw spot forming cell counts (SFC) from ELISpot and Fluorospot assays were converted and analyzed as SFC per 10^6^ PBMC (this reflects the number of spot forming cells per 10^6^ PBMC). Duplicate and triplicate SFC values were averaged for a given condition. SFC/10^6^ PBMC values from the negative control (culture media and DMSO) from each assay was subtracted from the signal from each peptide pool or single peptide stimulation. PHA was used as a positive control. SFC/10^6^ PBMC values above 100 were considered positive.

### 2.9 Statistical analysis

P values displayed in 2x2 tables were derived using Fisher’s exact test. Statistical analyses performed to compare IPV and TESLA ranking metrics were done using Prism 9 (GraphPad, La Jolla, CA). Mann Whitney tests were conducted and results were considered significant at p-values ≤ 0.05 (*).

## 3 Results

### 3.1 Generation of epitope candidates for TESLA and IPV pipeline comparison

**Figure 1:**
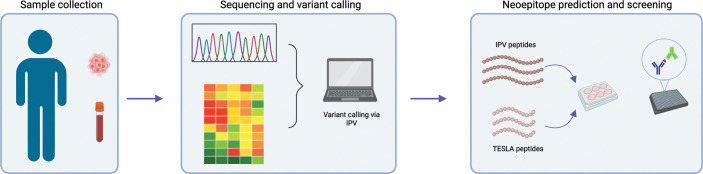
Overview of project workflow. Schematic outlining the workflow of the project. Whole blood and tissue were isolated from each patient. Blood samples were used for whole exome sequencing and tumor samples were used for exome and RNA sequencing. IPV was used to identify highly expressed, tumor-specific mutations from this sequencing data. The prioritized mutations were then used to generate neoepitope candidates either using IPV or TESLA ranking metrics. Finally, epitope candidates were cultured *in vitro* with patient PBMC and an IFNg and IL-5 fluorospot was used to assess reactivity.

PBMC and tumor tissue were obtained from 11 HNSCC patients. DNA was isolated from blood samples and both DNA and RNA were isolated from the tumors and used for sequencing as outlined in the methods. Whole exome sequencing (WES) and RNA sequencing from patients’ whole blood and tumor tissue were used to identify somatic variants present in each patient (**Figure 1**). We used our in-house pipeline to detect somatic variants and considered all expressed variants for this study (as described in the methods). We identified a median of 372 (min-max = 196-990) tumor associated variants per patient which is within the range of what has been reported in the literature (**Supplementary Table 1**) (32, 33). These variants were then filtered for highly expressed mutations in the tumor as described before (24). Using an in-house pipeline, the filtered mutations were translated to amino acid sequences in order to identify neoepitope candidates to be tested with autologous PBMC *in vitro* (29). We used two *in silico* epitope prediction methods to generate neoepitope candidates: our in-house pipeline, IPV, and the TESLA pipeline (19, 24).

The two pipelines differ in the way neoepitope predictions are generated. The IPV pipeline uses patient exome sequencing data to identify all nonsynonymous mutations. Following this, it ranks somatic variants based on variant allele frequency (VAF) in DNA and RNA and expression levels of source genes as measured in transcript per million (TPM) in the tumor. The IPV pipeline then generates two 20mer peptides for each mutation with the mutation at positions 6 and 15 (as detailed in the methods section). In contrast, the TESLA pipeline does not provide guidelines for detecting and ranking variants. Instead, the TESLA pipeline requires a list of predetermined somatic variants as an input. From the variants, the TESLA pipeline generates all possible 8-12mers and ranks the peptides per the TESLA metrics as published in the publication and implemented by us as described in the methods (19). Here, we used the IPV and TESLA pipelines to identify epitope candidates to test. We used the IPV pipeline for the somatic mutation identification step (which is lacking from TESLA), and then applied both IPV and TESLA to generate a set of peptides to test as epitope candidates.

### 3.2 Experimental testing of epitope candidates shows that IPV identifies immunogenic peptides at higher frequency than TESLA

To compare the predicted epitope candidates by the two pipelines, we tested their ability to activate autologous PBMC from cancer patients *in vitro.* The top ten peptides identified by each pipeline for each of the patients were selected for *in vitro* screening. Of the peptides selected for testing, 9 of the epitopes identified by TESLA were also found within the sequences of 20mer peptides identified by IPV. The remaining epitope sequences did not overlap between the two pipelines (**Supplementary Table 3**).

**Figure 2:**
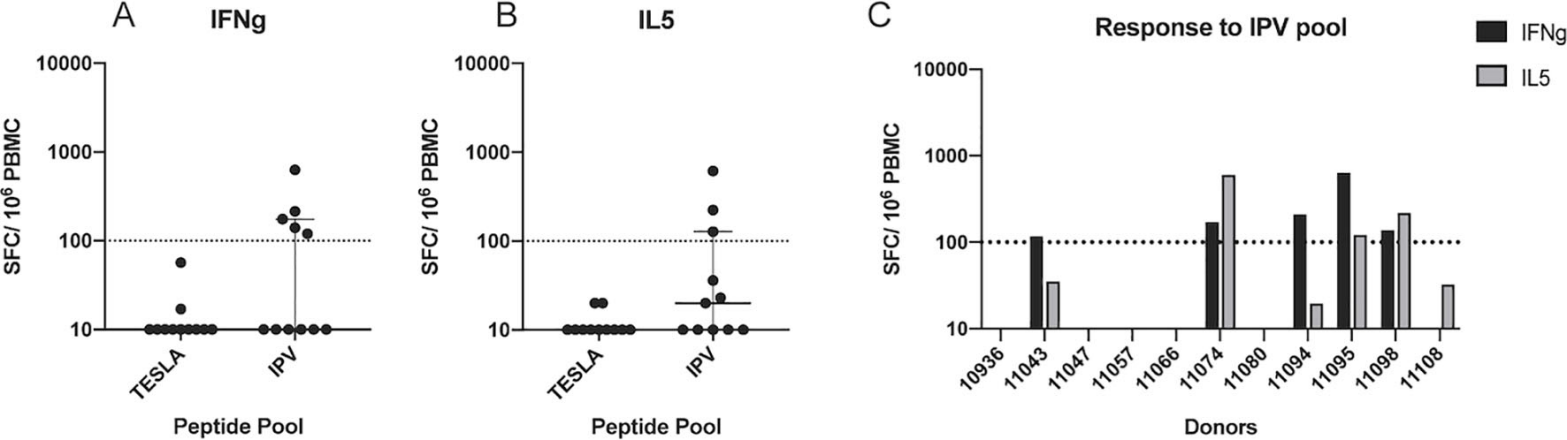
Systematic comparison of the immunogenicity of IPV and TESLA peptides. **(A)** The magnitude of the IFNg response from patient PBMC (one dot represents one patient) to neoepitope pools containing the top 10 TESLA and IPV peptides. The magnitude is represented as the number of spot-forming cells (SFC) per 10^6^ PBMC. A threshold of 100 SFC (dotted line) is used to distinguish between positive and negative responses. **(B)** IL-5 fluorospot data represented as SFC per 10^6^ PBMC. The same threshold as above is applied for the IL-5 data. **(C)** IFNg and IL-5 fluorospot-detected responses to IPV peptides from each patient within the cohort. IFNg responses are displayed as black bars and IL5 responses as grey bars.

Patient PBMC were stimulated with the candidate neoepitope pools and expanded in culture for 14 days *in vitro*, and then re-stimulated with the same peptide pools. Following expansion and re-stimulation, a dual IFNg/IL-5 FluoroSpot was used to determine the frequency of cytokine secreting cells in response to the pools of epitope candidates and thus quantify their immunogenicity. This culture and assay format was established for the sensitive detection of low frequency T cell responses (34). In the cancer setting, the primary readout is IFNg, secreted by CD8+ T cells and Th1 cells, but inclusion of IL-5 as a second cytokine comes with only minor additional costs in the Fluorospot assay and can provide insights on the presence of epitope specific Th2 cells. Strikingly, the TESLA peptides did not yield IFNg responses by any PBMC (**Figure 2A**). However, 5 out of the 11 patient PBMC cultured with IPV epitope candidates had a positive response for IFNg (**Figures 2A, C**). In addition, no PBMC secreted IL-5 more than the background level when stimulated with TESLA peptides (**Figure 2B**). In contrast, 3 out of 8 patients had a frequency of IL-5 producing cells above the background level following stimulation with IPV peptides (**Figures 2B, C**). Thus, the IPV pipeline identified a significantly higher fraction of peptide candidates that induced cytokine secretion by patient PBMC.

### 3.3 Peptide length is an important determinant of neoepitope immunogenicity

To determine the role that peptide length played in the outperformance of TESLA peptides (which are 8-12mers, “TESLA short”) by IPV (which are 20-mers, “IPV long”), two additional pools were generated: one pool of 20mers from the TESLA pipeline and one pool of 8-12mers from IPV (**Table 1**). NetMHCpan was used to generate 8-12mers from the 20mer IPV peptides by selecting the top predicted HLA class I binders from the 20mers (“IPV short”). The top 10 peptides were selected to generate this pool. Overlapping 20mer peptides generated by the IPV pipeline that contained the 8-12mers from the TESLA pipeline were used to generate the pool of TESLA 20mers (“TESLA long”). The top 10 20mers for each patient were selected based on the rank of the original 8-12mer in the TESLA pool. Due to the limited number of PBMC from each patient, we prioritized testing the TESLA short and IPV long peptides (i.e., the original peptide length for the pipelines) in each donor and tested the additional pools when enough PBMC were available. We were able to test the IPV short pool in 4 donors and the TESLA long pool in 8 donors. The 14-day expansion method outlined previously was used to screen both of the additional pools.

**Table 1:** Description of peptide pools generated for each patient.

Pool	Peptide length	Description
TESLA short	8-12mers	Original TESLA output
IPV short	8-12mers	Best predicted binders from IPV pipeline
TESLA long	20mers	Lengthened TESLA peptides
IPV long	20mers	Original IPV output

**Figure 3:**
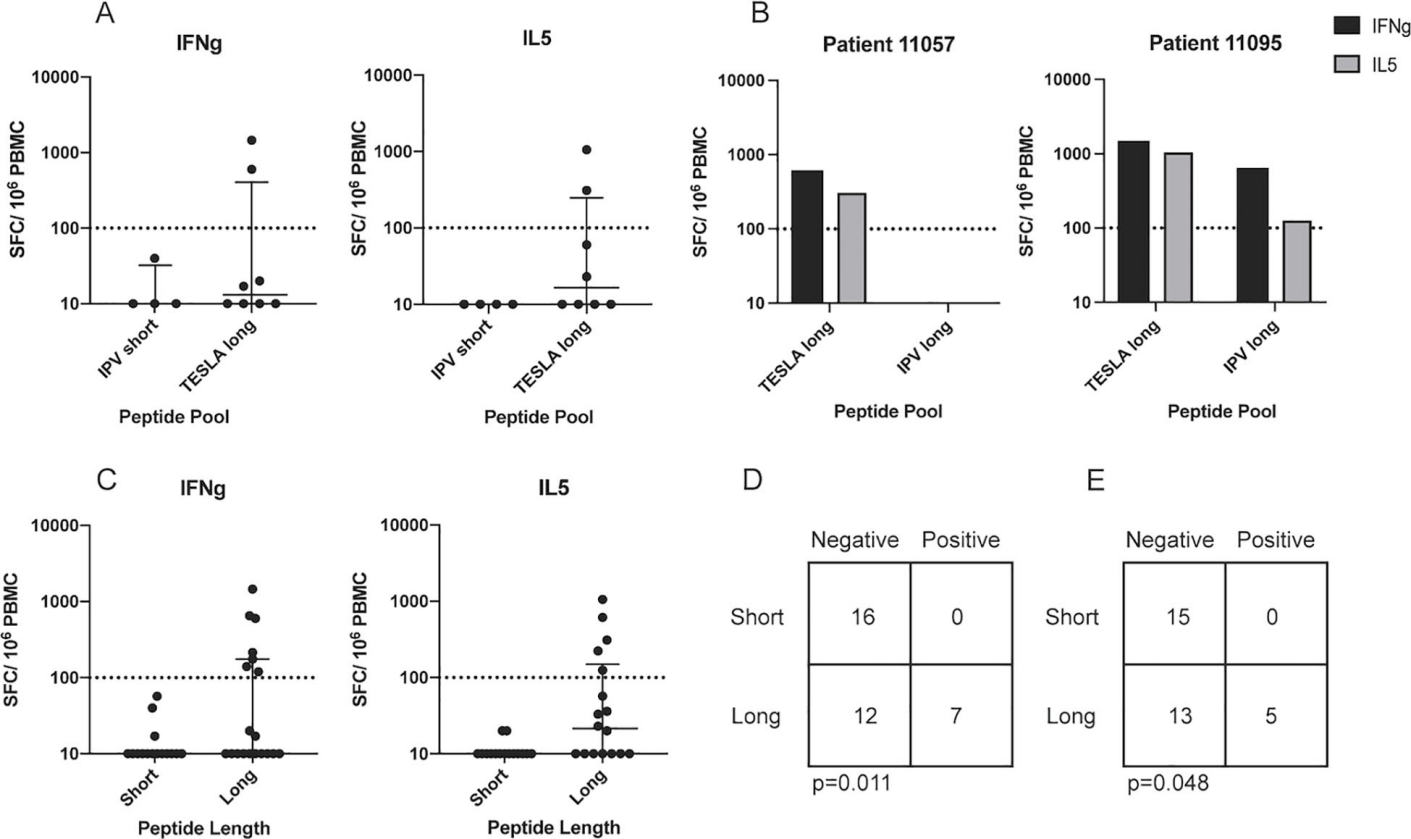
Peptide length plays an important role in peptide *in vitro* immunogenicity. **(A)** The magnitude of the IFNg and IL5 responses from patient PBMC to neoepitope pools containing the top 10 IPV short and TESLA long peptides. The magnitude is represented as the number of spot-forming cells (SFC) per 10^6^ PBMC. A threshold of 100 SFC (dotted line) is used to distinguish between positive and negative responses. **(B)** The magnitude of IFNg and IL5 responses from patients 11057 and 11095 PBMC in response to stimulation with the TESLA long and IPV long pools. **(C)** IFNg and IL5 fluorospot data from short and long neoepitope candidate pool stimulation. IFNg responses are displayed as black bars and IL5 responses as grey bars. **(D)** 2x2 contingency table comparing the performance of short and long peptides for IFNg. **(E)** 2x2 contingency table comparing the performance of short and long peptides for IL5.

None of the 4 PBMC cultures stimulated with the IPV short pool yielded positive IFNg or IL5 responses (**Figure 3A**), whereas 2 of the 8 PBMC cultures, from patients 11057 and 11095, secreted IFNg and IL5 in response to TESLA long peptide stimulation (**Figure 3A**). While PBMC from donor 11057 only responded to the TESLA long pool, PBMC from patient 11095 had positive IFNg and IL5 responses as a result of stimulation with both the TESLA long and IPV long pools (**Figure 3B**). Interestingly, both pipelines prioritized neoepitope candidates from the gene ATR for patient 11095 and thus the two pools both contained the ATR 20mers. In contrast, there was no overlap between the peptides included in the TESLA and IPV long pools for patient 11057 which may have contributed to the lack of response to the IPV long pool in this donor.

The Fluorospot data from the 14-day stimulation of the PBMC from our cohort of 11 patients with the four peptide pools were grouped and re-stratified based on the peptide length to compare the responses originating from long peptides (20mers) versus short peptides (8-12mers). A total of 7 IFNg and 5 IL-5 positive responses were detected from long peptide stimulation, while no positive responses were detected from stimulation with short peptides (**Figure 3C**). Thus, for both IFNg and IL-5, irrespective of the pipeline used to generate them, long peptides significantly outperformed the short peptides (Fisher’s test p-value=0.011 for IFNg and 0.048 for IL-5, **Figures 3D, E**). Taken together, this suggests that peptide length is a key determining factor for *in vitro* immunogenicity of epitope candidates in the experimental system used in our study. As a result of this finding, and our previous finding that the IPV long pool generated a greater frequency of cytokine positive responses compared to the TESLA long pool (**Table 2**), we solely focused on the IPV long pool for the remainder of this study.

**Table 2:** Summary of fluorospot data from the first cohort of patients.

		IFNg response		Il5 response		Percent positive		Performance comparison to TESLA short pool	
Pool	Number of patients tested	Positive	Negative	Positive	Negative	IFNg	IL5	IFNg	IL5
**TESLA short**	11	0	11	0	11	0%	0%	N/A	N/A
**IPV short**	4	0	4	0	4	0%	0%	p = 1	p = 1
**TESLA long**	8	2	6	2	6	25%	25%	p = 0.16	p = 0.16
**IPV long**	11	5	6	3	8	45%	27%	p = 0.035	p = 0.21

Positive and negative fluorospot responses from the neoepitope screen. The percent positive column was calculated by dividing the number of positive responses for a given pool by the number of times the pool was tested and representing that value by a percentage. The improvement in performance by a given pool in comparison to the TESLA short pool was calculated by performing a two tailed fisher’s test comparing the positive and negative responses of a pool to the TESLA short pool.

### 3.4 Deconvolution of positive IPV responses identifies neoepitopes that elicit cytokine responses

**Figure 4:**
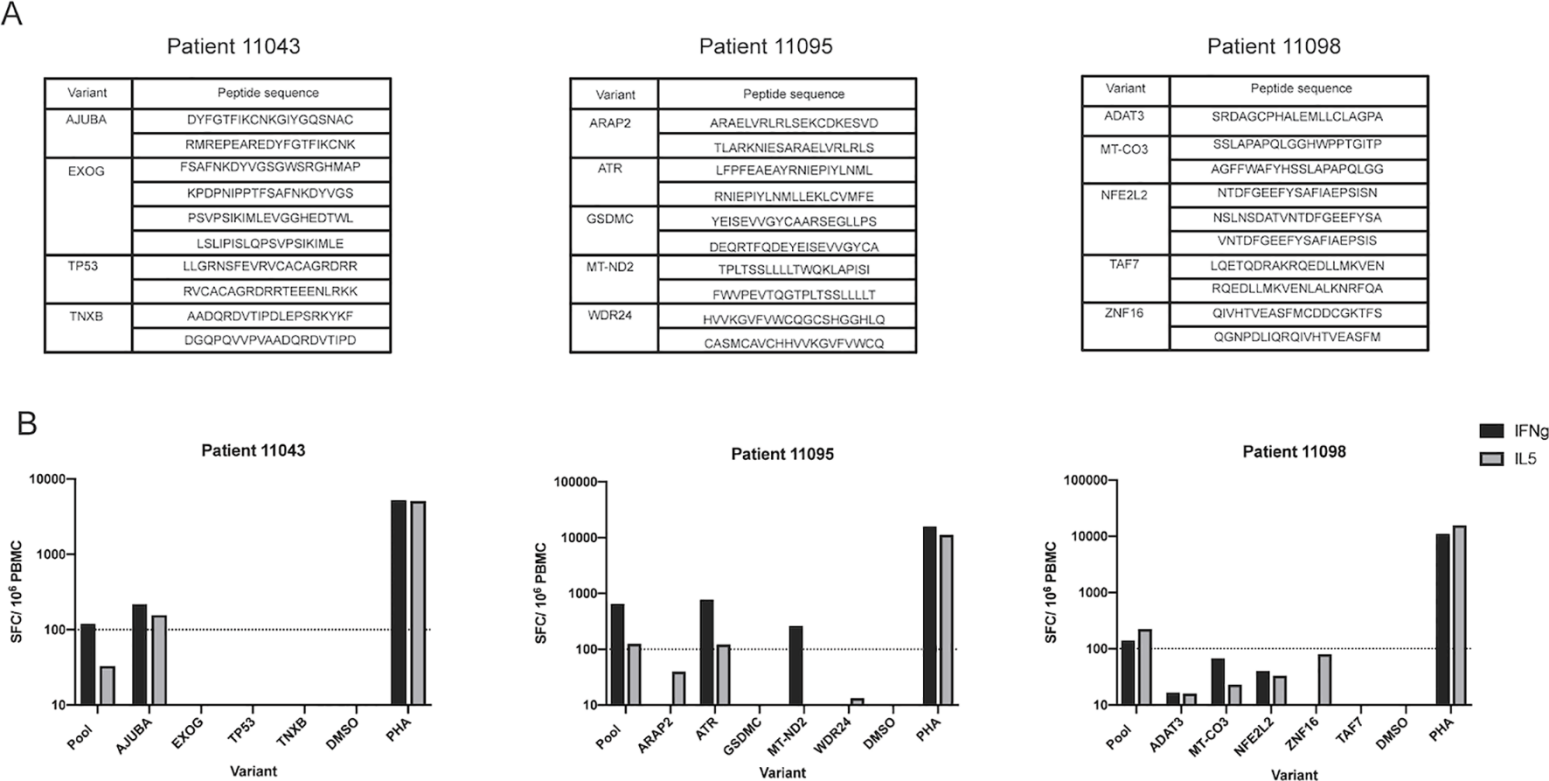
IPV long peptide pool deconvolution identifies immunogenic variants. **(A)** List of peptides generated from each somatic variant for patients 11043, 11095, and 11098. **(B)** Positive responses from the IPV peptide pool for patients 11043, 11095, and 11098 were deconvoluted to identify the variants contributing to IFNg and IL-5 signals. Magnitude of responses are displayed as SFC per 10^6^ PBMC for both IFNg and IL-5.

A subset of cancer patient PBMCs that showed positive responses to IPV long peptides and had a sufficient number of remaining PBMC was utilized to deconvolute the peptide pool signal. PBMC from three of the five patients (patients 11043, 11095, and 11098) who responded to IPV (**Figure 2C**) were thus used for deconvolution experiments. Following a 14-day expansion with the patient-specific peptide pools, PBMCs were re-stimulated with the peptides from each somatic variant included in the IPV pools to identify the somatic variants that were predominantly inciting cytokine release (peptide pools listed for each patient in **Figure 4A**). These deconvolution experiments allowed us to determine that peptides from the Ajuba LIM protein (AJUBA) variant were responsible for the positive cytokine response measured in patient 11043’s PBMC (**Figure 4B**). Additionally, epitope candidates from mitochondrial NADH dehydrogenase 2 (MT-ND2) and the serine/threonine-protein kinase ATR from patient 11095 were responsible for the positive response measured in patient 11095’s PBMC (**Figure 4B**). Interestingly, the 20mer peptides from the ATR mutation that elicited a response in PBMC from patient 11095 included the sequence of a 9mer ATR peptide identified by the TESLA pipeline (**Supplementary Table 3**). However, the PBMC from patient 11095 did not significantly respond to the TESLA peptide pool that included this peptide despite the response induced by the 20mer epitope. Additionally, the 20mer ATR peptides were included in both the TESLA long and the IPV long peptide pools. This may have contributed to the positive responses detected from both pools for this patient (**Figure 3B**). None of the peptides from individual somatic variants within patient 11098’s peptide pool sufficiently stimulated the PBMC on their own (**Figure 4B**). In conclusion, the IPV pipeline is able to provide insights into the immunogenicity of neoepitope peptide pools in cancer patients’ PBMC and identify the individual somatic variants that are responsible for this response.

### 3.5 IPV ranking metrics can successfully rank immunogenic epitope candidates higher than non-immunogenic epitope candidates

**Figure 5:**
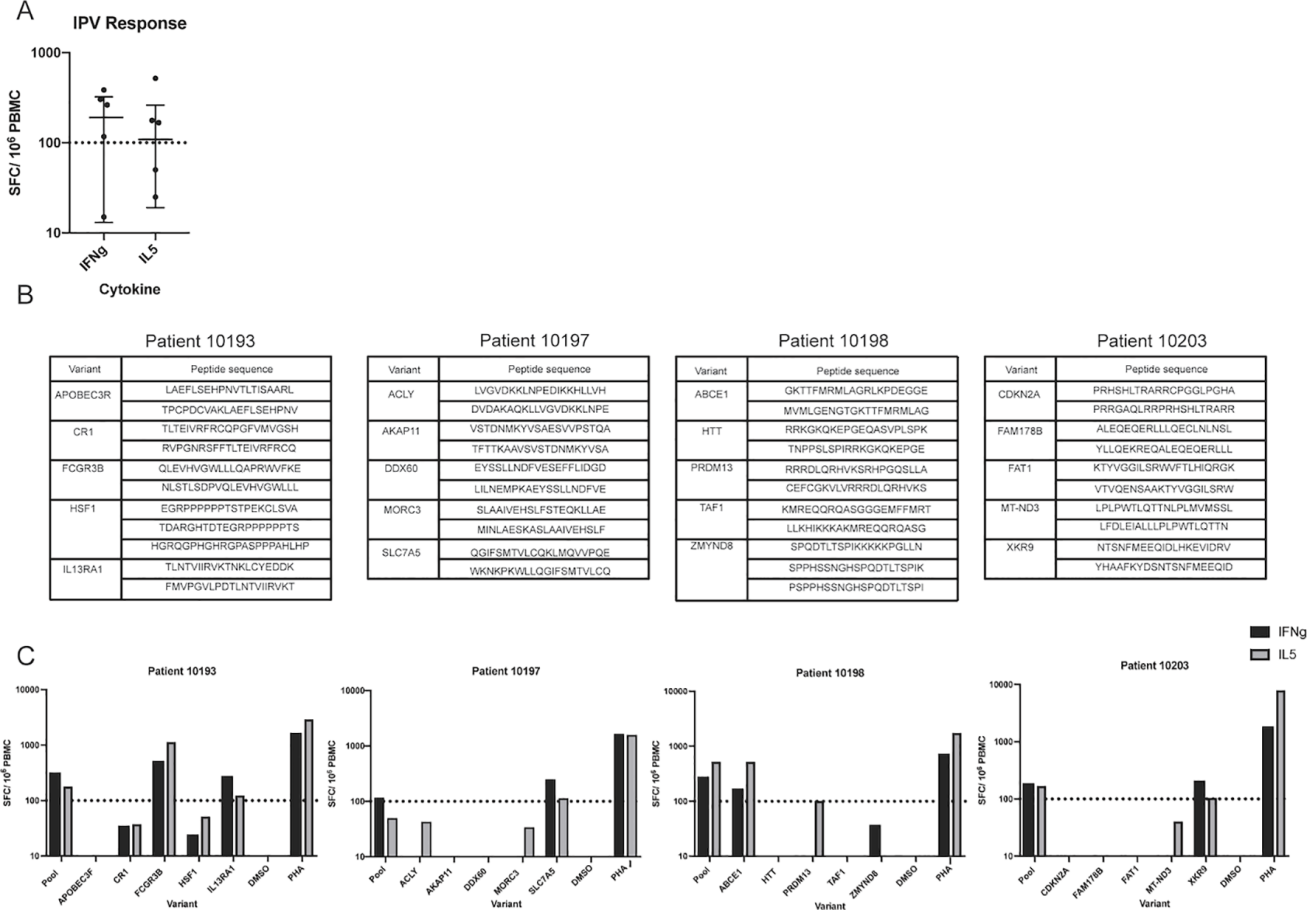
Application of IPV to an additional cohort of patients identifies additional immunogenic variants. **(A)** IFNg and IL-5 ELISpot was used to detect responses to IPV peptides in a second cohort of HNSCC patients (n=6) (one dot represents one patient). **(B)** List of peptides from each somatic variant used to deconvolute the second cohort responses. **(C)** Deconvolution data for patients 10193, 10197, 10198, and 10203.

To more broadly assess what variables are associated with specific immunogenic peptides, we needed more peptides identified than those from the 3 individuals described above. We thus applied the IPV pipeline to an additional cohort of six HNSCC patients. Whole blood and tumor samples were obtained from each patient and, similarly to our previous cohort, the IPV pipeline was used to identify somatic variants and predict neoepitope candidates from exome and RNA sequencing. Patient PBMC were cultured with IPV pools of the top ten epitope candidates (20mers) per patient, similarly to the first cohort of patients. After 14 days, PBMC were re-stimulated with the same epitope pool initially used for expansion, and an IFNg and IL-5 ELISpot was used to measure cytokine release. 4 of the 6 patients had a positive IFNg response and 3 patients had a positive IL-5 response following stimulation with IPV peptide pools (**Figure 5A**). Additionally, the four positive responses (patients 10193, 10197, 10198, and 10203) were deconvoluted as described previously (list of peptides outlined in **Figure 5B**). Patient 10193 PBMC were activated by peptides encoded by interleukin 13 receptor alpha 1 (IL13RA1) and Fc-gamma receptor 3B gene (FCGR3B) variants, patient 10197 responded to peptides from solute carrier family 7 member 5 (SLC7A5), patient 10198 responded to peptides from ATP binding cassette subfamily E (ABCE1), and patient 10203 responded to peptides from XK related 9 (XKR9) (**Figure 5C**). Thus, our IPV pipeline was successfully applied to an additional cohort of patients and was able to identify, for each of the patients, individual cancer neoepitopes that showed *in vitro* reactivity.

**Figure 6:**
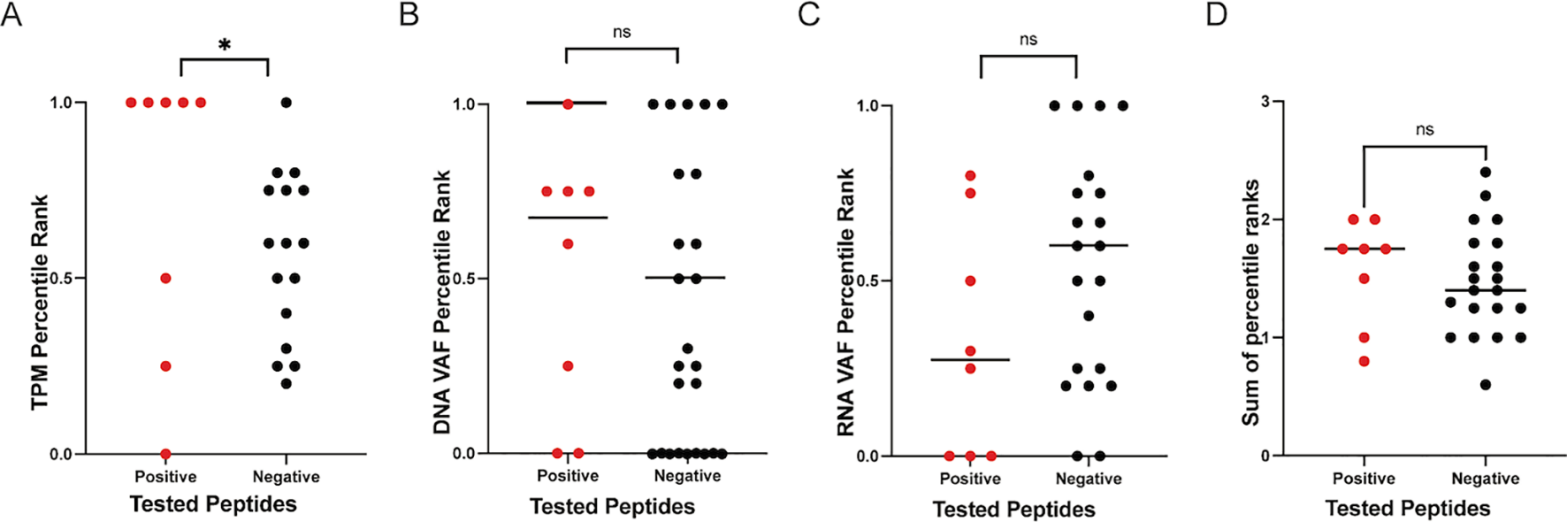
Ranking metrics and epitope immunogenicity. The percentile ranks for TPM, DNA VAF, and RNA VAF values for all peptide candidates were calculated on a per donor basis. The percentile ranks for the ranking metrics employed by IPV to rank positive epitopes higher than negative epitopes was assessed. The TPM **(A)**, DNA VAF **(B)**, RNA VAF **(C)** and sum of all metrics **(D)** for all the variants tested in this study are plotted. One dot represents one variant. ‘*’ indicates a p-value less than 0.05. ‘ns’ indicates non-significant p-values.

We analyzed all the tested variants from both patient cohorts to assess the performance of the individual ranking metrics included in the IPV pipeline for their ability to accurately rank immunogenic epitopes (i.e., those that incited a positive cytokine response *in vitro*) higher than the epitope candidates that were not immunogenic (i.e., those that did not induce a cytokine response *in vitro*). TPM, DNA VAF, and RNA VAF are the metrics used by IPV. When investigating the TPM percentile rank of the epitopes, the TPM rank placed 5 of the 8 positive epitopes higher than the negative epitopes and the median of the positive epitopes’ TPM percentile rank was significantly higher than the median of the negative epitopes (**Figure 6A**). Neither the DNA VAF or the RNA VAF had a significant difference between the positive peptides and the negative peptides, and summing the ranks together for each epitope also did not significantly distinguish positive epitopes from negatives (**Figures 6B–D**). This suggests that the TPM expression level of a given mutation had the strongest impact on the immunogenicity of the peptide encoding it in this setting.

As a comparison, we also investigated how effective the TESLA metrics were in prioritizing positive epitopes. HLA typing was solely performed on the samples from the first cohort of patients, so the TESLA metrics were only compared for the epitopes tested from this cohort since HLA typing is required for the TESLA pipeline. The HLA binding affinity of the positive epitopes was stronger (i.e., lower IC50) than the negative epitopes, but the difference was not significant. In contrast, there was no difference in the agretopicity or stability of the positive and negative epitopes and the foreignness scores of multiple negative epitopes were higher than the foreignness of the positive (**Supplementary Figure 2**). The finding that the HLA binding affinity is stronger in positive epitopes than negative epitopes is not surprising as previous work by many groups, including ours, has shown that the majority of neoepitopes have strong predicted binding affinities (35). The analysis of the agretopicity, stability, and foreignness of the epitopes, however, does not support that these metrics contribute to the ability of distinguishing between positive and negative epitopes.

## 4 Discussion

In this study, we compared the performance of our in-house pipeline, IPV, and the TESLA pipeline to predict the immunogenicity of neoepitope candidates present within HNSCC patients with limited blood sample available. Our results highlight the challenges intrinsic to such comparisons, and the importance of considering the experimental system used to evaluate immunogenicity predictions. The features included in the TESLA prediction pipeline were picked based on an evaluation of neoepitope prediction pipelines in their performance, where pMHC-multimer assays using minimal HLA class-I restricted epitopes were used to evaluate immunogenicity and establish the ground truth (19). The identification of minimal epitopes with defined HLA-restriction is however not necessary to test peptides for T cell immunogenicity. Moreover, the TESLA approach ignores the potential contribution of HLA class II restricted epitopes to anti-tumor responses. In a cancer setting, limited blood samples and limited time make it desirable to have a fast and sensitive approach to screening, which informed our development of IPV. IPV ranks somatic mutations based on their level of expression and their tumor association (i.e. the expression of a given mutation in a tumor sample compared to a normal sample) due to previous literature that suggests that the magnitude of expression of a particular antigen significantly correlates with its ability to be recognized (36). From the ranked variants, IPV then generates 2 overlapping 20mers per variant to allow for all possible class I and class II epitopes to be investigated rather than predicting minimal epitope sequences using HLA typing and HLA binding predictions. Due to the significant differences in the two approaches, we sought to compare the performance of the TESLA pipeline to IPV and determine which of the two is the more appropriate pipeline to use.

Here, we report that the in-house IPV pipeline significantly outperformed TESLA in the ability to predict epitope candidates that stimulate cytokine release by autologous PBMC *in vitro.* From the first cohort of patients, the IPV pipeline 20mers (IPV long) elicited IFNg responses in about half of the patient PBMC and IL5 responses with 3 patients’ PBMCs while no PBMC secreted IFNg or IL-5 in response to stimulation by any of the TESLA short peptides. We hypothesized that peptide length (i.e., having longer peptides that can include both class I and class II restricted epitopes) played a role in this finding, and thus we investigated how peptide length influenced the *in vitro* activation of PBMC by candidate peptides. We found that no short peptides (neither TESLA short nor IPV short) generated IFNg responses while long peptides (either TESLA long or IPV long) generated IFNg responses 7 of the 18 times they were tested). Overlapping long peptides that encompass both class I and class II peptides have been shown to generate strong T cell responses in numerous studies and many groups have reported that anti-tumor immunity is highly dependent on the presence of CD4 T cells (37–39). Interestingly, a small subset of TESLA epitopes were derived from mutations that were also prioritized by the IPV pipeline (**Supplementary Table 3**). In the case of patient 11095, both TESLA and IPV predicted epitopes from a single nucleotide variant in the gene ATR. The 20mer epitope predicted by IPV encompassed the 9mer predicted by TESLA. However, only the IPV 20mer elicited an immune response. This highlights the benefit in generating overlapping 20mers, as IPV does, as it eliminates the possibility of prioritizing the wrong minimal epitope sequence for a given mutation and provides the opportunity to activate CD4+ T cells. In consideration of the results from other groups as well as the positive cytokine responses in our study, it becomes evident that in addition to HLA class I epitopes, HLA class II epitopes must also be considered in *in silico* neoepitope prediction tools.

To further study responses to IPV, we screened neoepitope candidates in an additional cohort of 6 HNSCC patients using the IPV pipeline and deconvoluted the positive responses from both cohorts of patients. Deconvolution from the first cohort identified that peptides from the mutations in AJUBA activated PBMC from patient 11043 and peptides from ATR and MT-ND2 activated PBMC from patient 11095. Peptide pool deconvolution in the second cohort successfully identified that neoepitopes from the variants IL3RA1, FCGR3B, SLC7A5, ABCE1, and XKR9 activated PBMCs from patient 10193, 10197, 10198, and 10203, respectively. Importantly, elevated expression and mutations in these genes have been reported to promote tumor cell growth and inhibit apoptosis in various cancer types (40–44). For example, ATR is a serine/threonine kinase that is involved in sensing DNA damage and mutations in this kinase contribute to the genomic instability of cancer cells (45, 46). Additionally, mutations in ND2 (the subunit of mitochondrial NADH dehydrogenase), have been shown to confer increased metastatic potential to cancer cells (47). The immunogenicity of these variants serves as evidence that our pipeline can effectively parse through patient somatic data and prioritize mutations that are biologically relevant and may have therapeutic benefits.

Although we show here that IPV can be used to identify immunogenic overlapping peptides, we did not identify the minimal epitope sequences of the immunogenic peptides. However, because our pipeline can identify 20mer peptides that are immunogenic, one can use the IPV pipeline and various available computational tools to predict the minimal epitope sequence if that is of interest. For example, one could use the sequences of IPV 20mers that induce cytokine responses in a given patient and the patient’s HLA typing and use tools such as TepiTool to predict the HLA binding affinity of the possible minimal epitopes to a patient’s HLA alleles (48). With this information, one could conduct immunogenicity assays with the minimal epitope sequences that have strong predicted HLA binding affinity. Overall, the IPV pipeline could serve as an excellent first step in identifying the exact sequences of immunogenic epitopes while conserving the amount of patient sample needed.

Finally, we found that the percentile rank of the TPM of the variants, a feature considered by the IPV pipeline, can successfully rank the variants whose peptides induced cytokine responses (positive epitopes) higher than those that did not (negative epitopes). The percentile ranks for both TPM and DNA VAF effectively ranked positive epitopes higher than negative epitopes, although the difference in DNA VAF was not significant. Interestingly, RNA VAF ranked the positive epitopes lower than the negative epitopes. The low RNA VAF in some of the recognized epitopes could be a result of a number of factors. Firstly, this could be indicative of a tumor undergoing immune-editing to evade immune recognition. Tumors rely on immune-editing to deplete the expression of neoepitopes that are being recognized by immune cells (49). Additionally, this could indicate a low purity tumor sample or a genetically diverse tumor (50). The sum of the ranking metrics employed by IPV appropriately ranked positive peptides higher than negative peptides, but this difference was not statistically significant. TPM and VAF are influenced by the depth and quality of the sequencing methods employed to detect tumor antigens. Additionally, all the neoantigens selected for this study ranked highly for these metrics, which may be the cause for the lack of a statistically significant difference between positive and negative epitopes. None of the TESLA metrics were able to discern statistically significant differences between positive and negative peptides. While our work suggests that our selection approach is effective, additional research is necessary to further identify and understand the features that differ between positive and negative peptides and the features that are useful for neoepitope prediction tools.

There are several caveats to our study. First, we have only compared the IPV pipeline to the TESLA pipeline, but a number of additional pipelines and ranking features exist. For example, several tools take into account the biophysical properties of epitope candidates to predict which sequences are more likely to contribute to strong TCR-peptide interactions (51, 52). This shortcoming can be addressed in future studies in which we provide further comparison of our pipeline to other tools that focus on alternative features. An additional limitation of our study is that the experimental method we used to compare the pipelines could influence the types of responses that are more easily detected. Here, we used a 14-day expansion method in which patient PBMC were cultured with neoepitopes candidates for 14 days and fed with IL-2 on days 4, 7, and 10. Subsequently, we utilized a IFNg and IL-5 Fluorospot or ELISpot to measure the amount of cytokine release occurring as a result of stimulation with epitopes. The amount of cytokine detected via Fluorospot and ELISpot is thought to correlate with epitope immunogenicity and antigen-specific responses (53). We elected to use this method as it circumvents the need to know the exact sequences of HLA-restricted epitopes and allows for the expansion of low-frequency memory cells in peripheral blood samples. However, alternative methods to test neoepitope recognition exist. For instance, peptide-MHC multimers can be used to detect neoepitope-specific T cells as was done by Wells et al. (19) This experimental method presents biases for certain HLA alleles and works better for peptides with shorter length and known exact epitope sequences (54, 55). The use of tandem minigenes (TMG) is also a method that is routinely used to screen antigens. In this method, multiple antigen candidates are strung together in a single DNA or RNA construct to be processed and presented by antigen-presenting cells to T cells. While TMGs allows for multiple antigen candidates to be screened at once in an HLA-independent manner, it has been recently suggested that the order of sequences within minigenes can influence screening outcomes (56, 57). Thus, the experimental method elected by a researcher could influence the results of neoepitope screening. Therefore, this should be taken into consideration in studies moving forward and highlights the need for clear experimental standards to be used when evaluating neoepitope prediction tools.

Our findings strongly suggest that IPV is a pipeline that can be used to predict the neoepitopes that generate robust T cell responses *in vitro* and that the pipeline described by TESLA is not as effective in this experimental setting. The outperformance of TESLA by IPV is a significant finding as the approach used in the TESLA pipeline (i.e. ranking class I peptides based on HLA binding affinity, agretopicity, HLA binding stability, and foreignness) are metrics that have been routinely included in a number of neoepitope prediction tools (51, 58–61). Furthermore, our study provides a comprehensive pipeline that can be used to identify neoepitope candidates from patients with a limited sample size.

## Data Availability

Fluorospot data presented in the study are available in the supplementary data. Whole exome and RNA sequence data are deposited in the BioProject repository, accession number PRJNA1229652.
