# Supplementary material for: Comparative performance analysis of neoepitope prediction algorithms in head and neck cancer
Source: Front Immunol. 2025 Mar 4;16:1494453. doi: 10.3389/fimmu.2025.1494453 (PMC11914794; doi:10.3389/fimmu.2025.1494453)
Supplement: Supplementary file 1 [file DataSheet1.zip › Supplementary Information/Supplementary_Material.docx]

Supplementary Material

#
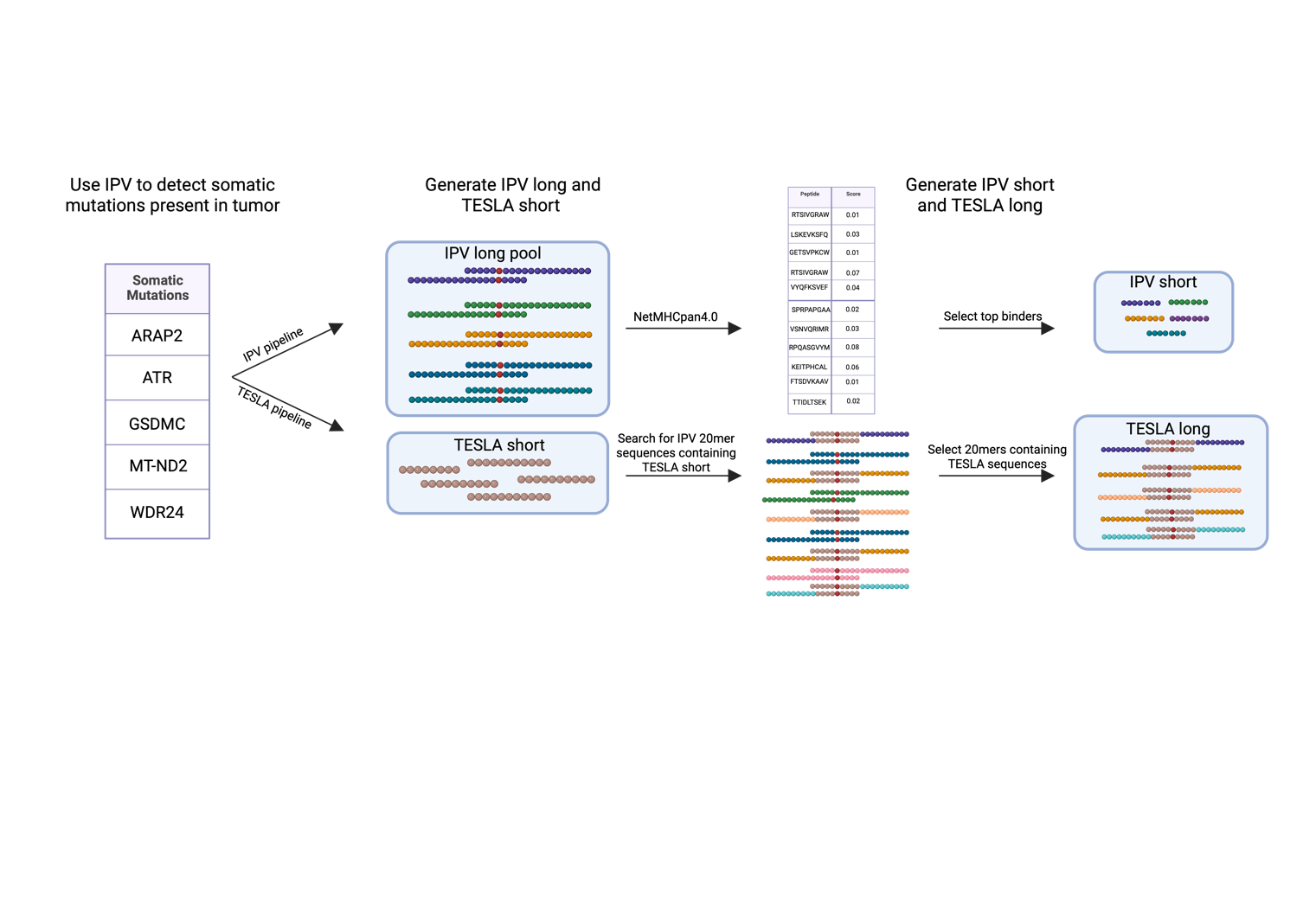
Supplementary Figures

**Supplementary Figure 1. Overview of neoepitope generation**

Schematic outlining the process of generating neoepitope candidates from a given somatic mutation as described in the methods.

**Supplementary Figure 2. Analysis of TESLA metrics ability to rank epitope candidates**

The MHC binding affinity (A), MHC binding stability (B), Agretopicity (C) and Foreignness (D) for all the variants tested in this study are plotted.

# Supplementary Tables

**Supplementary Table 1. Number of somatic variants identified by IPV**

| **Patient ID** | **Number of variants** |
| --- | --- |
| 10936 | 990 |
| 11043 | 223 |
| 11047 | 410 |
| 11057 | 314 |
| 11066 | 472 |
| 11074 | 250 |
| 11080 | 515 |
| 11094 | 364 |
| 11095 | 196 |
| 11098 | 372 |
| 11108 | 465 |

**Supplementary Table 3. TESLA peptide sequences found in IPV 20mer peptide sequences**

| **IPV peptide sequence** | **TESLA peptide sequence** | **Patient** | **Gene name** |
| --- | --- | --- | --- |
| RQEDLLMKVENLALKNRFQA | LMKVENLAL | 11098 | TAF7 |
| NSLNSDATVNTDFGEEFYSA | NTDFGEEFY | 11098 | NFE2L2 |
| NTDFGEEFYSAFIAEPSISN | NTDFGEEFY | 11098 | NFE2L2 |
| VNTDFGEEFYSAFIAEPSIS | NTDFGEEFY | 11098 | NFE2L2 |
| INSNKTNIRFMEPLYMFCAM | RFMEPLYMF | 11094 | LRRN1 |
| VLCSVSPSGSRVPHNLLGGS | SPSGSRVPHNLL | 11057 | FAAP100 |
| NFYQSYGPSGQYTHEFDGDE | SYGPSGQYTHEF | 10657 | HLA-DQA2 |
| LVMSYVLLPAAMVMPPQPVV | SYVLLPAAM | 11057 | TOLLIP |
| PPLPQDTPFFPGQPIPPHEF | TPFFPGQPI | 11080 | PCED1A |
| LFPFEAEAYRNIEPIYLNML | YRNIEPIYL | 11095 | ATR |
| PSGQYSHEFDGDEEFYVDLE | YSHEFDGDEEFY | 10657 | HLA-DQA2 |
